# Bilateral gamma knife radiosurgery in severe essential tremor: a prospective trial

**DOI:** 10.1093/brain/awag231

**Published:** 2026-07-23

**Authors:** Jean Régis, Valentin Mira, Serge Pinto, Eve Benchetrit, Corentin Gaillard, Jeremy Danna, Lauriane Veron-Delor, Axelle Cretol, Silvia Sandu, Sarah Fernandes, Jean-Philippe Azulay, Alexandre Eusebio, Marianne Vaugoyeau, Tatiana Witjas

**Affiliations:** Institut Neurosciences Systèmes (INS), INSERM, Aix Marseille University, Marseille 13284, France; Department of Stereotaxis, Assistance Publique Hôpitaux de Marseille (AP-HM), Functional Neurosurgery & Radiosurgery Marseille 13385, France; CNRS, CRPN, Aix Marseille University, Marseille 13003, France; Department of Neurology and Pathology of Movement, Timone Hospital, Marseille 13385, France; CNRS, LPL, Aix Marseille University, Aix-en-Provence 13100, France; Department of Neurology and Pathology of Movement, Timone Hospital, Marseille 13385, France; Department of Neurology and Pathology of Movement, Timone Hospital, Marseille 13385, France; CNRS, CLLE, Toulouse University, Toulouse 31058, France; CNRS, CeRCA, Poitiers University, Tours University, Poitiers 86073, France; Department of Stereotaxis, Assistance Publique Hôpitaux de Marseille (AP-HM), Functional Neurosurgery & Radiosurgery Marseille 13385, France; Department of Stereotaxis, Assistance Publique Hôpitaux de Marseille (AP-HM), Functional Neurosurgery & Radiosurgery Marseille 13385, France; CERESS, Aix Marseille University, Marseille 13385, France; Department of Biostatistic, Aix-Marseille University, Marseille 13385, France; CNRS, CRPN, Aix Marseille University, Marseille 13003, France; Department of Neurology and Pathology of Movement, Timone Hospital, Marseille 13385, France; Department of Neurology and Pathology of Movement, Timone Hospital, Marseille 13385, France; Institut Neurosciences Timone (INT), CNRS, Aix Marseille University, Marseille 13005, France; CNRS, CRPN, Aix Marseille University, Marseille 13003, France; Department of Neurology and Pathology of Movement, Timone Hospital, Marseille 13385, France

**Keywords:** essential tremor, gamma knife radiosurgery, thalamotomy, bilateral treatment, VIM

## Abstract

In patients with medically refractory essential tremor, unilateral Gamma Knife radiosurgery (GKS) of the ventral intermedius thalamic nucleus (VIM) is known to improve contralateral tremor with good safety. Safety of staged bilateral GKS of the VIM has never been strictly evaluated. Our aim was to determine whether staged bilateral VIM-GKS is safe and effective, avoiding side effects associated with bilateral surgery, while providing improvement in tremor impairment.

We conducted a prospective, single-arm, phase 2 trial of second-side GKS of the VIM in 33 patients with severe essential tremor and no relevant speech or balance problems. A comprehensive assessment before surgery, as well as 6 and 12 months post-surgery, included complete neuropsychological testing, the evaluation of voice, speech, gait, posture, tremor severity, activities of daily living, disease-related quality of life and MRI. The primary end point was safety.

All patients completed the study after the 1-year follow-up period (19 males, 14 females, 32 right VIM-GKS and 1 left). A single adverse event was observed (hemi-proprioceptive ataxia and dysarthria due to hyper-response 11 months after VIM-GKS). The mean delay between the first and the second GKS was 28.7 months. The postoperative cognitive assessment, as well as the evaluation of speech, gait and balance, showed no significant worsening. The tremor severity score significantly improved by 58.5%, the disability score by 84.8% and the functional impact score by 68.6%. Four patients failed to respond, while the remaining 29 patients showed a mean improvement of 74.4% in their treated upper limb tremor. Quality of life at 12 months was significantly improved.

In selected patients with bilaterally disabling essential tremor and without speech, gait or balance concerns, staged bilateral VIM-GKS can be performed with a good safety profile and leads to a major reduction in tremor and improvement in quality of life. If confirmed by larger series and longer follow-up, bilateral staged VIM-GKS could be considered as a good option for selected patients presenting with a severe drug-resistant bilaterally disabling essential tremor.

## Teaser

With MR-guided focused ultrasound and radiosurgery, we are witnessing a renaissance of ablative treatments for drug-resistant essential tremor. Régis *et al.* report the first prospective phase 2 trial of staged bilateral radiosurgery, demonstrating tremor reduction with no significant worsening of cognition, speech, gait or balance.

## Introduction

Essential tremor (ET) is the most frequent movement disorder, with a prevalence of about 1/200.^1,2^ ET causes social disability and difficulties in activities of daily living (ADL), which worsen with time and lead to progressive loss of autonomy. Treatment such as beta-blockers, primidone or antiepileptics might have some efficacy at the beginning. However, as the severity of the tremor increases, efficacy tends to decrease in 30%–50% of patients.^2^ The VIM (ventral intermedius) thalamic nucleus is the most commonly used neurosurgical target for treatment of drug-resistant ET. In the pre-magnetic resonance stereotactic neurosurgery era, bilateral radiofrequency thermocoagulation of the VIM (VIM-RF) was reported to induce specific severe side effects, including dysarthria, hypophonia, gait and balance issues, and cognitive impairment.^3^ Since then, bilateral VIM surgery has been considered at high risk of complications. In the 1990s, bilateral deep-brain stimulation of the VIM (VIM-DBS) was demonstrated to be safer than VIM-RF and became the gold standard.^4^ However, long-term tolerance, gait/balance and speech complications have been reported after bilateral VIM-DBS, in addition to a significant rate of tremor recurrence.^5^ Gamma Knife radiosurgery (GKS) is a minimally invasive neurosurgical procedure, presenting the advantage of not requiring skull opening and introduction of electrodes into the brain. It reduces the risk of bleeding and infection and minimizes the risk of mistargeting by avoiding brain-shift. Unilateral VIM radiosurgery (VIM-GKS) has demonstrated a good safety and efficacy profile in ET,^6-8^ with no worsening of gait and balance.^9^ Some use of staged bilateral VIM-GKS has been reported but only in retrospective studies.^10^ Thus, the aim of our prospective trial was to evaluate the safety and efficacy of staged bilateral VIM-GKS.

## Materials and methods

### Study design and patient selection

We conducted a monocentric, prospective, single-arm trial of staged bilateral VIM-GKS in patients presenting with severe ET, according to the Movement Disorder Society Consensus Statement criteria.^11^ Between 2010 and 2018, patients who had previously undergone unilateral VIM-GKS at our institution and sought contralateral treatment were screened for eligibility (Fig. 1).

**Figure 1:**
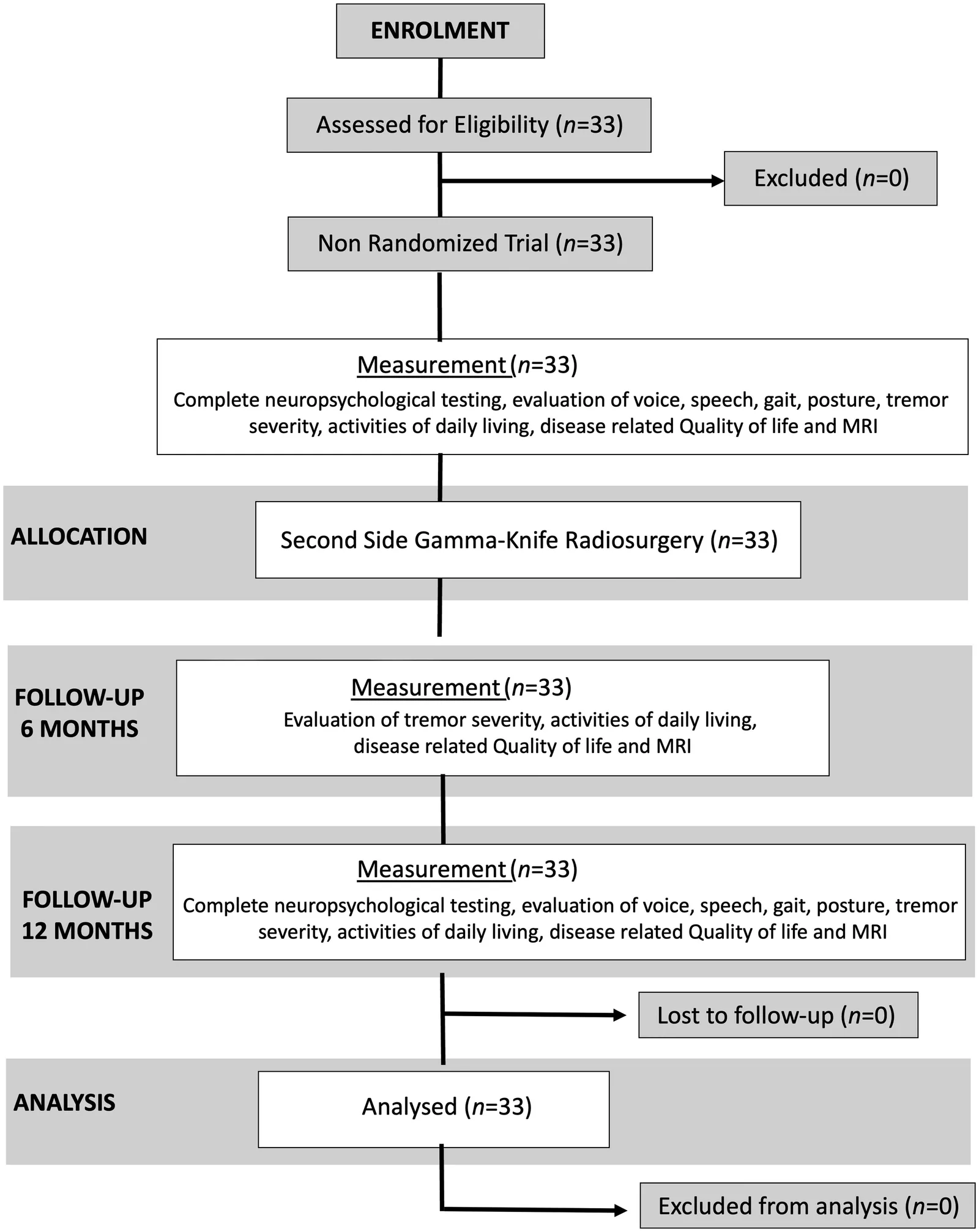
**Flow chart of the study**.

We included all the patients who had undergone unilateral VIM-GKS for the dominant hand at least 1 year before the second surgery, presented with a good outcome [>45% improvement in tremor severity score; and/or >50% improvement in ADL; and no side effects], and considered the tremor of the non-dominant hand severely disabling (self-reported and based on scores).^6^ Patients with significant gait and posture impairment (defined as altered gait with more than two missteps in tandem walking) with disabling dysarthria/speech problems (dysarthria score > 2) and or cognitive decline [Mini-Mental State Examination (MMSE) score ≤ 24] were excluded (Supplementary material 1).

### Radiosurgical procedure

Patients enrolled in the trial were treated using the Perfexion or Icon Gamma Knife radiosurgical device (Elekta Instruments), in line with our previously published procedure.^6,12^ Notably, a diffusion tensor imaging (DTI) sequence was performed, and after frame application, stereotactic MR and CT scan imaging were acquired. A single isocentre of 4 mm was positioned at the location of the VIM according to the ‘Guiot scheme’, with adjustment of the target based on the preoperative DTI, according to our usual standard technique (Supplementary material 8). The VIM-GKS procedure was similar for both surgeries, and no changes to the dose prescription or targeting were made. All the interventions were performed by the same neurosurgeon (J.R.).

### Assessments

#### Safety

Assessments of cognitive functions, gait/balance and voice/speech were performed before surgery and 12 months after, in order to investigate side effects classically reported after bilateral VIM surgery.

A thorough neuropsychological testing included the MMSE, Trail Making Test part B, similarities (Wechsler Adult Intelligence Scale III), verbal fluencies (semantic and lexical), Stroop Test (interference), Visual Object and Space Perception Battery (letters and numbers), Starkstein Apathy Scale, Barratt Impulsiveness Scale and Free and Cued Selective Reminding Identifies Test (FCSRT—Encoding Phase, FCSRT—Total Free Recall, FCSRT—Delay Free Recall, FCSRT—Delay Total Recall).

Kinematic gait analysis was performed with the SMART TV image processing system (eMOtion). Speed, cadence, step length, step width, coefficient of variation and Tandem (number of missteps) were calculated. The AMTI force platform was used for balance assessment. For further details, see Supplementary material 3.

The speech/voice assessment included the self-evaluation of the patients’ impairment with two dedicated questionnaires (Voice Handicap Index, Dysarthria Impact Profile) and the audio recording of speech and non-speech oromotor tasks (maximal phonation time, MPT; sustained vowel/a/; oral diadochokinesis; short text reading), which allowed for *a posteriori* calculation of several acoustic variables as recommended in the most recent guidelines.^13^ Speech intelligibility was evaluated by rating single-word and sentence production and using the perceptual score of the French Clinical Evaluation Battery of Dysarthria (Batterie Clinique d’Evaluation de la Dysarthrie, BECD). Speech intelligibility and perceptual assessments were performed after the last post-operative examination of the last patient. The raters were blinded to the pre- or post-surgery status of the recordings, which were presented randomly. For further details, including the variables measured, see Supplementary material 4.

#### Efficacy

Tremor assessment, quality of life and MRI were performed before and at 6 and 12 months after the second VIM-GKS. Tremor severity score was assessed using the Fahn-Tolosa-Marin (FTM) rating scale and Whighet scale.^6^ Functional disability was assessed using the Bain and Findley Tremor Activities of Daily Living scale and the FTM part C. Tremor severity was assessed under medication.

We used the Quality of Life in Essential Tremor (QUEST)^14^ score, which is an ET-specific, validated, patient-reported quality-of-life assessment.

The MRI assessment included measurement of the volume of T1 contrast enhancement on the side treated by the second procedure, as well as T2 hypersignal and oedema score at 6 and 12 months (Fig. 2).

**Figure 2:**
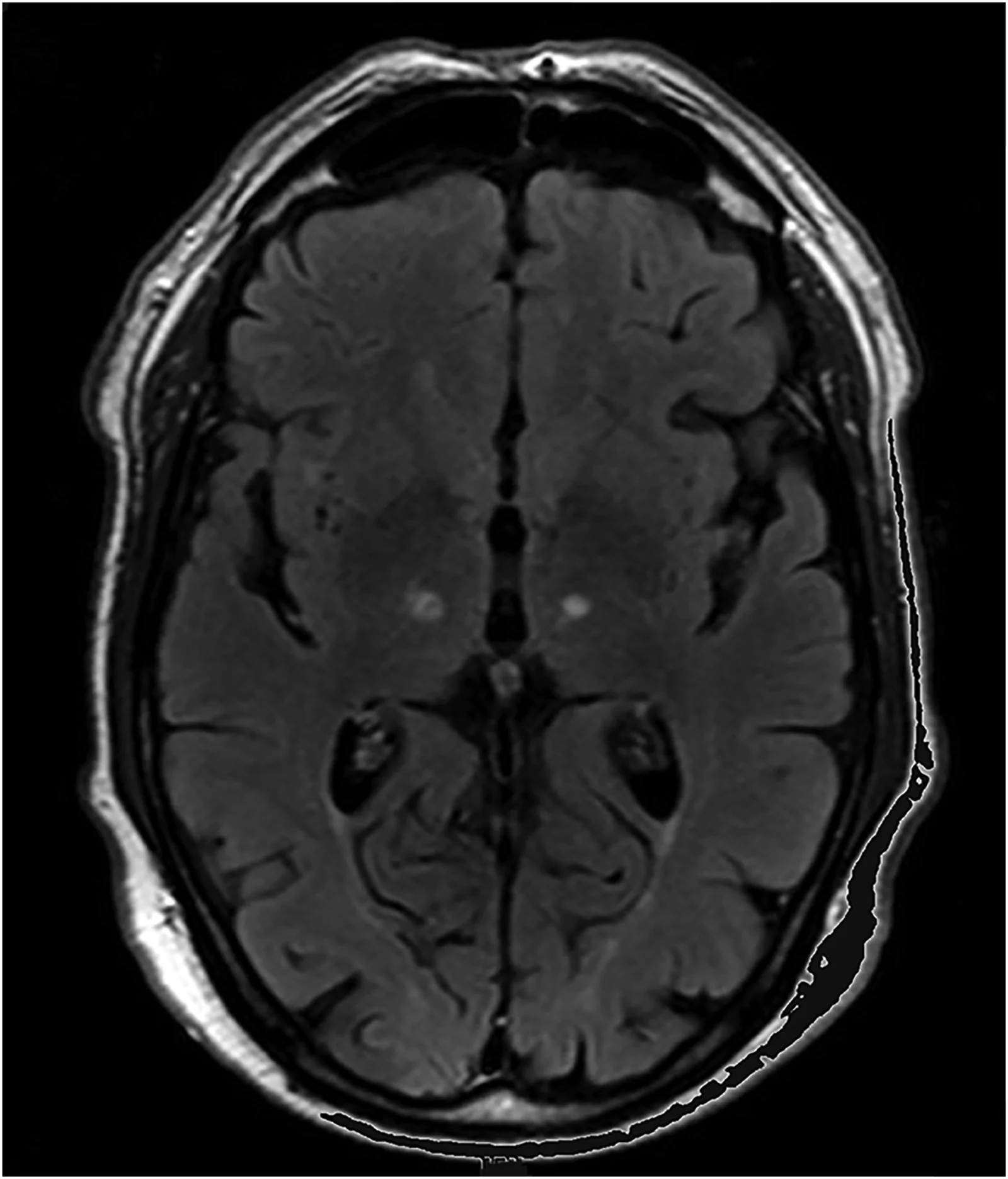
**MR image at 2 years demonstrating the high T2 intensity at the level of the VIM nucleus bilaterally and symmetrically**. VIM = ventral intermediate nucleus of the thalamus.

### Statistical analyses

Frequencies and percentages were considered for categorical variables, mean ± standard deviation (SD) for normally distributed continuous variables, and median and interquartile range (IQR) for non-normally distributed continuous variables. Depending on data distribution, continuous variables were compared between baseline and 12 months using paired *t*-tests or Wilcoxon signed-rank tests. Generalized estimating equation (GEE) models were used to assess changes over time for repeated measures at three time points (baseline, 6 and 12 months after GK2). A two-sided *P*-value <0.05 was considered statistically significant. Statistical analyses were performed using IBM SPSS, version 20.0 (SPSS Inc., Chicago, IL, USA).

### Ethical aspects

This study was approved by the local ethics committee and registered under EUDRACT/IDRCB: 2013-A01289-36 before enrolment of the first patient. All patients provided written informed consent. The results were followed and reviewed by an independent Data and Safety Monitoring Board (Marwan Hariz and Paul Krack).

## Results

### Patients

A group of 33 patients [19 men, 14 women; mean age: 71 years, median age: 70 years (67–75), (range 55–83)] was included in this study. All had previously been treated for tremor of the dominant hand (32 for the right hand and 1 for the left). None of these patients experienced any side effects after the first surgery. Treatment of the second side was performed with a median delay of 24 months (IQR 20–28; range 16–74) between the first and second radiosurgeries. All 33 patients completed the study at the 1-year follow-up. Patient characteristics are summarized in Supplementary material 2. The median VIM-GKS treatment time was 75 min (53.5–121.575 min).

### Safety

One serious adverse event was observed in one patient. The patient presented at 8 months with rapid reduction of left upper limb tremor, and at 11 months, proprioceptive ataxia of the left upper limb and dysarthria appeared, with the MR demonstrating greater contrast enhancement than usual. See the MR picture of this hyperresponse in Supplementary material 6. Thanks to rehabilitation, the dysarthria improved dramatically in <3 months. The speech assessment at 12 months showed good intelligibility, but self-perceived impairment (VHI) was slightly increased. Upper limb ataxia improved partially, but at the last follow-up (9 years), the patient was still walking with the assistance of one person. The long-term side effects were moderate. She considered her QUEST to be 34 before the GK2 and 30 at 1 year after GK2.

Regarding cognitive functions, all assessments demonstrated no change post-surgery (Table 1). A similar trend was observed for the gait and balance quantitative assessment, which showed no significant worsening post-surgery (Table 2). Postural evaluation before and 1 year after treatment, in the natural and Romberg conditions, revealed no significant differences in any of the parameters. The patients’ subjective perception of their ability to walk was stable, and no fall was reported by any patient 1 year after the second VIM-GKS.

**Table 1:** Cognitive function before radiosurgery on the second side and at 12 months

Test		Median (IQR)	*P*-value
MMSE	Baseline	28 (27–29)	Reference
	12 months	29 (27–29.5)	0.448
Trail Making Test, part B	Baseline	25 (23–25)	Reference
	12 months	25 (24–25)	0.1609
WAIS similarities, part III	Baseline	10.50 (8.50–12.50)	Reference
	12 months	10 (8–11.75)	0.2893
Verbal fluencies, semantic	Baseline	0 (0–1)	Reference
	12 months	0 (0–1)	0.977
Verbal fluencies, lexical	Baseline	0 (0–1)	Reference
	12 months	0 (0–1)	0.964
Stroop test	Baseline	0.480 (0.395–0.565)	Reference
	12 months	0.445 (0.335–0.590)	0.531
VOSP- letters	Baseline	20 (19–20)	Reference
	12 months	20 (18–20)	0.227
VOSP-numbers	Baseline	9 (8–10)	Reference
	12 months	9 (7.50–9.50)	0.389
Starkstein Apathy Scale	Baseline	8 (4–11)	Reference
	12 months	9 (5–13)	0.481
Barratt Impulsiveness Scale	Baseline	66 (57.50–72)	Reference
	12 months	60 (51–70)	0.246
FCSRT—encoding phase	Baseline	16 (14–16)	Reference
	12 months	16 (15–16)	0.4725
FCSRT—total free recall	Baseline	23 (19–27.25)	Reference
	12 months	25 (20.25–30)	0.1580
FCSRT—total recall	Baseline	46 (42.75–48)	Reference
	12 months	46 (41–47.75)	0.7346
FCSRT—delay free recall	Baseline	8 (6–10)	Reference
	12 months	9 (7–11)	0.2015
FCSRT—delay total recall	Baseline	16 (14–16)	Reference
	12 months	15 (14–16)	0.9476

FCSRT = Free and Cued Selective Reminding Identifies Test; IQR = interquartile range; MMSE = Mini-Mental State Examination; VOSP = Visual Object and Space Perception Battery; WAIS = Wechsler Adult Intelligence Scale.

**Table 2:** Gait assessment and postural assessment before radiosurgery on the second side and at 12 months

	Parameter	Median difference	Pairs, *n*	*W*	*P*-value	Different
**Walking condition**						
Natural	Speed (mm/s^2^)	39.8	30	−1	0.99	No
	Cadence (step/min)	3	30	81	0.42	No
	Step length (mm)	−9	30	−93	0.35	No
	Step width (mm)	1.14	30	77	0.44	No
	Cov of step length	−9.3	30	−93	0.35	No
Tandem	Number of missteps	0	28	36	0.57	No
**Postural condition**						
Natural	Path	−1.03	24	−76	0.28	No
Tandem	PeT score	1.65	29	−24	0.32	No

Statistical results of gait performance. Median difference = difference between pre- and post-VIM-GKS with the pre-condition as reference. Statistical results of postural evaluation after equivalence testing. Median of difference = difference between pre- and post-VIM-GKS with the pre-condition as reference. CoV = coefficient of variation; PeT = Postural evaluation of Tremor; *W* = Wilcoxon.

Patients’ self-assessments of speech/voice showed no difference between the pre- and post-operative states (Voice Handicap Index: *P* = 0.86; Dysarthria Impact Profile: *P* = 0.74). In addition, no difference was found for the sentence intelligibility rate; isolated word intelligibility rate was worse (*P* = 0.011) post-operatively, despite remaining at an average of 90%. Regarding acoustic variables, the Harmonics-to-Noise ratio (HNR) of the sustained vowel/a/was significantly better (*P* = 0.035) post-operatively (20.08) than pre-operatively (17.81). For all other acoustic variables, no pre- versus post-operative difference was found, except the articulatory rate of the oral diadochokinesis task, which demonstrated a significant difference (*P* = 0.02) between baseline and 1-year after surgery, the patients being slightly slower post-operatively (4.59 syllables/s) than pre-operatively (4.88 syllables/s). (Table 3 summarizes all speech/voice assessment variables; Supplementary material 4). Furthermore, neither postoperative self-reported dysphagia nor dysgeusia was observed.

**Table 3:** Speech and voice assessment before radiosurgery of the second side and 12 months after

Task measure	At VIM–GKS 2	12 months after	*P*-value
**Acoustics**			
Maximum sustained vowel/a/			
Maximal phonation time, s	15.4 (1.2–47.81)	16 (1.6–52.2)	0.681 ns
Oral diadochokinesis			
Articulatory rate (syllable number/speech duration), syllables/s	4.88 (3.07–6.86)	4.59 (1.80–6.45)	**0.020** ^a^
Speech proportion (total speech duration/total duration of the session), s	0.93 (0.69–0.99	0.94 (0.7–0.98)	0.726 ns
Pause proportion (pause duration/total duration of the session), s	0.07 (0.01–0.31)	0.06 (0.02–0.3)	0.734ns
Performance index (speech proportion/number of breath groups)	0.16 (0.03–0.5)	0.19 (0.08–0.49)	0.616 ns
Breath duration, s	6.19 (0.98–15.8)	6.61 (2.43–16.77)	0.544 ns
Pause duration, s	0.47 (0.25–3.30)	0.47 (0.28–2.92)	0.902 ns
Number of syllables	27.4 (3–85.5)	13.75 (4.36–72)	0.959 ns
Sustained vowel/a/			
Mean F0, Hz	137.84 (76.17–218.88)	141.63 (83.83–224.96)	0.245 ns
F0 SD, Hz	4.09 (0.92–52.4)	4.54 (0.86–21.2)	0.178 ns
Mean SPL, dB	73.99 (63.34–86.40)	73.05 (62.33–90.92)	0.483 ns
SPL SD, dB	1.47 (0.71–10.71)	1.73 (0.48–11.9)	0.382 ns
Harmonics-noise ratio	19.55 (3.5–25.2)	20.66 (9–34.3)	**0.035** ^a^
Jitter, %	0.006 (0.004–0.029)	0.006 (0.003–0.023)	0.492 ns
Shimmer, Hz	0.048 (0.19–0.2)	0.037 (0.012–0.16)	0.060 ns
**Speech intelligibility and perceptual assessment**			
Speech intelligibility section of the Frenchay Dysarthria Assessment			
Sentences (intelligibility rate), %	100 (86.67–100)	100 (53.3–100)	0.754 ns
Words (intelligibility rate) %	96.7 (80–100)	93.3 (36.67–100)	**0.011** ^a^
Perceptual clinical evaluation (BECD)			
Phonetic realization	1.50	2.26	**0.0** ^a^
**Self-reported speech impairment questionnaires**			
Voice handicap index			
Total score	8.27 (0–56)	9.12 (0–64)	0.845 ns
Physical subscore	1.50 (0–15)	1.50 (0–18)	0.810 ns
Functional subscore	0.50 (0–25)	0.50 (0–18)	0.861 ns
Emotional subscore	0 (0–18)	0 (0–28)	0.972 ns
Dysarthria impact profile			
Total score	176.62	176.38	0.980 ns

BECD = Batterie d’Evaluation Clinique de la Dysarthrie (i.e. Evaluation Battery for the evaluation of dysarthria); F0 = fundamental frequency; SD = standard deviation; SPL = sound pressure level.

^a^ *P*-values in bold refer to significant statistical comparison between pre- and post-surgery data.

No significant correlation was observed between T1 contrast enhancement volume and clinical improvement at 6 months (Spearman’s ρ = 0.068, *P* = 0.705) or 12 months (Spearman’s ρ = 0.044, *P* = 0.808). Similarly, volumes did not differ significantly between responders and non-responders at either time point (Supplementary material 7).

### Efficacy

Tremor control assessment (Supplementary material 5) showed significant efficacy of VIM-GKS (*P* <0.001). The median delay for tremor improvement was 4.25 months (mean 4.51; min 1; max 12; Q1–3; Q3–6). Evaluation at 1-year follow-up (Figs 3 and 4) demonstrated that the tremor severity, functional disability and impact on ADL mean scores improved by 59.2%, 67.7% and 84.4%, respectively. The median values and standard deviation (SD)/IQR are provided in Supplementary material 5. The tremor amplitude of the second side decreased by 71.5% at 12 months. Conversely, both ADL and amplitude of the first side scores did not change significantly at 12 months. Four patients failed to reach the 45% threshold for tremor score improvement, which appeared to be modest. For the 29 remaining patients, the mean improvement of tremor in the treated upper limb was 74.4% (range 35.3%–100%), and the median improvement was 76.4% (66.6%–87.5%).

**Figure 3:**
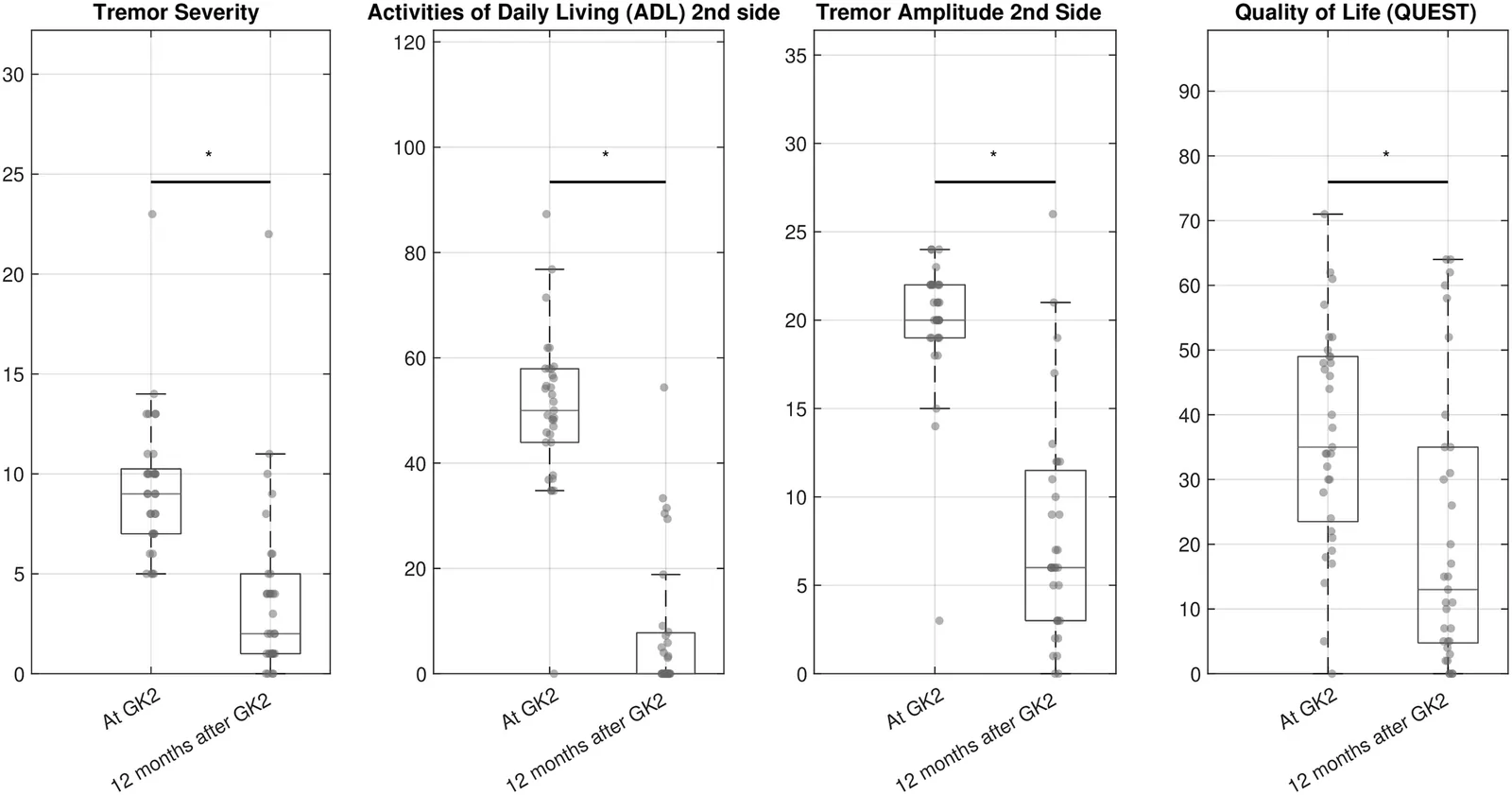
**Changes over time in overall tremor severity scores, functional disability scores, overall tremor amplitude (second side treated) and quality of life scores before GK2 and at 12 months**. QUEST = Quality of Life in Essential Tremor; GK2 = second Gamma Knife radiosurgery.

**Figure 4:**
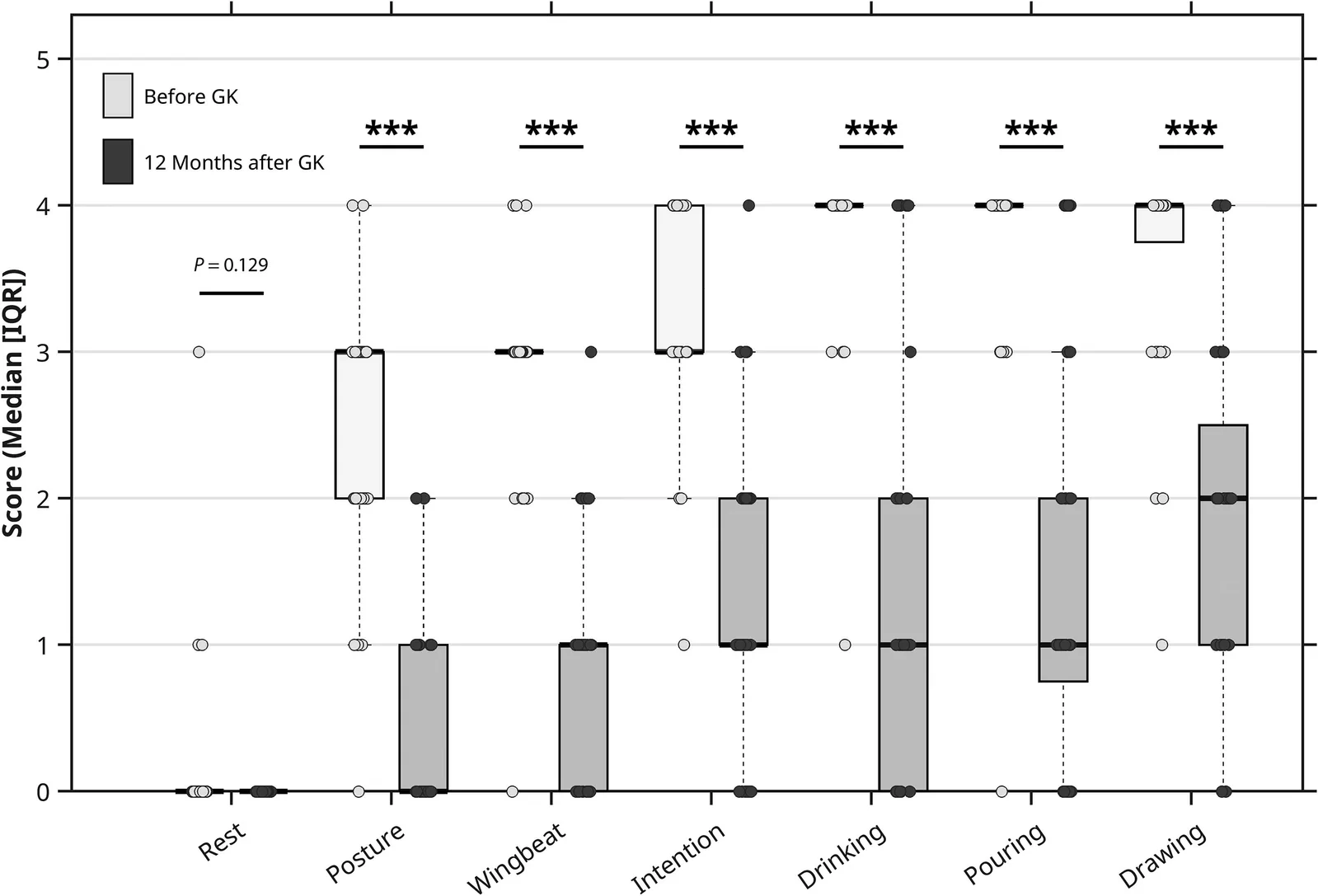
**Before and 12 months after GK2 comparison of the sub-scores for tremor assessments.** GK2 = second Gamma Knife radiosurgery.

Among the 13 patients presenting with a head tremor at inclusion, 10 (77%) showed an improvement of head tremor at 12 months: nine no longer exhibited head tremor, and one patient’s score improved from 3/4 to 2/4.

Quality of life (Fig. 3) was significantly improved at 12 months after the second side VIM-GKS (*P* < 0.001).

## Discussion

The aim of our original prospective study was to evaluate whether bilateral VIM-GKS, performed in a staged manner, would avoid side effects associated with bilateral surgery while providing improvement in tremor impairment. The neurosurgical dogma to treat patients unilaterally with VIM-GKS is largely due to the high historical rate of adverse effects reported when bilateral lesions of the thalamus were performed either simultaneously or in a staged manner.^3^ Permanent disabling complications, including dysarthria, hypophonia, proprioceptive ataxia, cognitive impairment and gait disturbances, were reported in up to 60% of VIM-RF thalamotomies.^15^ An additional reason for these high rates was that such procedures were performed using rudimentary stereotactic techniques, which have since been replaced by sophisticated targeting, possibly explaining the much lower rate of serious complications from staged bilateral RF thalamotomies in the paper by Horisawa *et al.*^16^

In this study, we demonstrated that staged bilateral VIM-GKS can be performed safely, avoiding severe side effects at 12 months post-surgery. Tremor scores and ADL were significantly improved by 58.5% and 68.6%, respectively, which is comparable to other surgical techniques such as magnetic resonance-guided focused ultrasound (MRg-FUS)^17^ and VIM-RF.^15^ Our results demonstrate no change of any neuropsychological assessment variables as well as gait, balance and posture at 12 months follow-up.

At the group level, we found no clinically significant voice/speech alteration after staged bilateral VIM-GKS. Slight changes in speech took the shape of reduced articulatory rate and an increase in perceptually assessed phonetic disturbances. The impact of radiosurgery on voice quality indicators was less conclusive, either negatively or positively. In fact, at the individual level, it is important to mention the high inter-individual heterogeneity of patient states at baseline and post-surgery outcomes. Globally, the patients’ self-perception of their voice/speech remained unchanged post-surgery, suggesting that such changes, when observed, did not represent a concern for the patients after bilateral VIM-GKS.

Nevertheless, speech/voice assessment exhibited a higher tolerance profile with VIM-GKS than with bilateral VIM-RF and VIM-DBS, which showed worsening in 13.9% and 34.6% of patients, respectively, after surgery.^18^ This is in line with a recent prospective bilateral trial of MRg-FUS performed in a smaller population sample showing a moderate negative dysarthria effect in 15% of the patients at 12 months.^19^

In a pivotal, prospective trial, Schuurman *et al.*^4^ opened a new era in which DBS was favoured over thalamotomy for ET treatment due to fewer adverse events, reversibility and adaptability.

We are now witnessing a renaissance of lesioning for tremor management with the development of MRg-FUS.^20^ Thus, the historical contraindications of bilateral lesioning of the VIM using VIM-RF, VIM-GKS or MRg-FUS again become a matter of debate when considering a paradigm shift for new, staged neurological procedures.

To our knowledge, there is no prospective study on staged, bilateral VIM-RF. In a retrospective study by Horisawa *et al.*,^16^ nine patients^17^ who underwent a staged bilateral VIM-RF surgery presented with persistent clinical adverse events, including dysarthria, dysgeusia, dysphagia, tongue numbness, unsteady gait and postural instability at the last follow-up.^16^ The authors considered these persistent adverse events to be mild and not to affect the patients’ daily activities.^16^ The only available observational cohort studies for bilateral, staged VIM-GKS reported improvement in some patients with no adverse events.^8,10^ Concerning staged MRg-FUS, several authors reported a safe profile for ET patients in three retrospective^21-23^ and two prospective trials.^19,24^

MRg-FUS is today one of the most popular options for tremor surgery, but several meta-analyses found no significant differences in efficacy or safety when comparing MRg-FUS and VIM-GKS.^20,25,26^ Interestingly, the spectrum of complications seems different: while the most frequent complication after VIM-GKS is a proprioceptive ataxia of the treated upper limb due to hyper-response to radiosurgery,^6^ gait and balance problems are more frequently observed after MRg-FUS. Elias *et al.*^17^ reported gait disturbances in 36% of patients after unilateral MRg-FUS, persisting in 9% of patients 12 months later. In a prospective trial assessing bilateral MRg-FUS for tremor published by Iorio-Morin *et al.*,^19^ despite stability of the quantitative metrics, the number of missteps during the 6-month tandem walk assessment worsened significantly. Additionally, two patients reported subjective gait impairment at 1 month, with falls. In a prospective multicentre trial, Kaplitt *et al.*^24^ reported mild, moderate and severe adverse events in 85%, 13% and 0.5% of patients, respectively, after bilateral MRg-FUS. The most commonly observed adverse events at 12 months were numbness/tingling (16%), dysarthria (14%), ataxia (12%) and dysgeusia (6%). The authors reported no clinically significant speech decline. Of note, the gait and balance assessment was limited to clinical examination with no quantified analysis of gait and balance. Conversely, in our cohort, we found no worsening of the quantitative metrics and tandem walk, and we observed no falls after bilateral VIM-GKS. Indeed, the mechanism of action of radiosurgery is completely different from that of acute thermocoagulation produced by RF and MRg-FUS. The precise delivery of a high dose of gamma rays in a small volume with a very steep fall-off of dose induces a focalized cascade of biological events, leading to a focal, small, necrotizing effect within a period of 4 months.^27^ Th specific action kinetics of VIM-GKS could provide an explanation for the absence of postural-locomotor impairment.

We hypothesize that this slow mechanism allows for the appearance of compensatory mechanisms involving the intact structures adjacent to the VIM.^9^ In the case of RF or MRg-FUS, the very acute thermocoagulation effect does not leave sufficient time for a compensatory mechanism, resulting in patients experiencing sudden but frequent transitory gait and balance impairments.^9^ Only in rare cases of ‘hyper-response’, the inflammatory messengers create cytotoxic oedema, disrupting these compensatory mechanisms, similar to the expansion to adjacent structures of the electrical field in supra-therapeutic stimulation.^28^ Despite having responded normally after treatment on the first side, it is difficult to draw a solid conclusion explaining why this patient developed a hyper-response after treatment of the second side, in the absence of any change in her medication. We think this is most likely due to the immune status of the patient at the time of the intervention rather than a genetic determinant.^29^ Interestingly, targeting was symmetrical in our study, unlike the Toronto bilateral MRg-FUS trial, which used a slightly different target on the second side, with the hope of lowering the risk of morbidity.^19^ Although VIM-GKS compares favourably with other techniques, it is less often used. One possible explanation is the absence of immediate clinical feedback or concerns regarding delayed hyper-response. For VIM-GKS, the neurosurgeon must rely heavily on their expertise in localizing the VIM on MRI and skillfully navigate the technical nuances of dose planning to ensure efficacy while minimizing the risk of hyper-response. Additionally, the requirement for combined expertise in both stereotactic functional neurosurgery and radiosurgery may represent a significant limiting factor.

In our experience of unilateral VIM-GKS, improvement of functional disability is observed in 74.2% of patients, with a mean delay of 4.5 months.^6^ Side effects occur in 7% of patients,^30^ usually taking the shape of a proprioceptive ataxia that induces a disability of the treated upper limb.^6^ However, in most patients, the treatment of the sole dominant hand provides a major improvement in disability.^31^ Additionally, it must be kept in mind that in bilateral VIM DBS, the adverse event profile can sometimes be corrected due to the adjustability of the stimulation parameters.^4^ Consequently, bilateral ablative techniques may be reserved for a selected group of patients.

## Conclusion

This first prospective trial on bilateral, staged VIM-GKS demonstrated a good safety profile and efficacy on tremor in a subgroup of selected patients with drug-resistant ET, previously treated unilaterally at least 1 year before the second intervention. The major benefit in terms of quality of life and functional independence was accompanied by no alteration in gait/balance, speech/voice or cognitive functions. Staged bilateral VIM-GKS appears to be a valuable option for the management of tremor within the available surgical armamentarium. Longer follow-up and larger cohorts are needed for further confirmation of our findings.

## Supplementary Material

awag231_Supplementary_Data

## Data Availability

Deidentified individual participant data underlying the published results and the study protocol will be available upon request. The request must come from an academic institution. Access requires prior approval by the sponsor, regulatory authorization and a data use agreement (contact: promotion.interne@ap-hm.fr). The data will be available immediately following publication and remain available for 5 years.
