# Supplementary material for: Bilateral gamma knife radiosurgery in severe essential tremor: a prospective trial
Source: Brain. 2026 Jul 23;149(10):3395–403. doi: 10.1093/brain/awag231 (PMC13634637; doi:10.1093/brain/awag231)
Supplement: Supplementary file 1 [file awag231_supplementary_data.pdf]

Supplementary Material 1 : Inclusion and exclusion criteria.

Inclusion criteria	
	Previous VIM-GKS on the dominant side more than 1 years before
	Age between 20 and 85 years
	Tremor severity score improvement ≥ 45% and or<br>Activity of Daily Living improvement ≥ 50%.
	No cognitive worsening (MMSE > 24)
	No severe gait and balance impairment (walk score ≤ 2)
	No dysarthria (Dysarthria score ≤ 2)
	Contrast enhancement ≤ 350mm3
	Informed consent understood and signed
Exclusion criteria	MR contra indication
	Previous radiotherapy of the head
	Pregnant or breast feeding women
	Inclusion in another trial
	Age under 20 or over 85
	MMSE score ≤ 24
	Dysarthria score > 2
	Walk score > 2

Supplementary Material 2 : Patient characteristics.

Parameter		Value
Sex, n(%)<br>Male<br>Female		 19 (57.6)<br>14 (42.4)
Age (years), median (IQR)		70 (67-75)
Disease duration before GK2 (months), median (IQR)		41 (9-72)
Age of onset of the tremor (years), median (IQR)		29.6 (5-60)
Past family history of tremor, n(%)		26 (78.8)
Delay since VIM-GKS 1 (months), median (IQR)		24 (20-28)
Transient side effect after VIM-GKS 1, n(%)		None
Permanent side effect after VIM-GKS 1, n(%)		None
Head tremor, n(%)		13 (39,4)
Primidone at the time of VIM-GK2, n(%)		9 (27.3)
Propranolol at the time of VIM-GK2, n(%)		12 (36.4)
Severity score improvement after VIM-GK1, median (IQR)		83.3 (20.8-100)
Functional disability score improvement after VIM-GK1, median (IQR)		89.0 (65-100)
MMSE score at baseline, median (IQR)		28 (25-30)
Walk score at baseline, median (IQR)		0 (0-0)
Dysarthria score at baseline, median (IQR)		0 (0-0)
T1 contrast volume on 1st side at VIM-GK1, median (IQR)		46 (16-82)

IQR : interquartile range.

Supplementary material 3 : Gait & posture

Gait

Patients performed walk test of a distance of eight metres at their natural speed and using tandem gait. During the tandem walk, patients were instructed to walk on a taped line and place their front foot such that their heel touched the toe of their back foot. In each condition, two trials were recorded. The mean of the two trials for each condition were used for the statistical analysis.

Kinematic gait analysis was performed with the SMART TV image processing system (eMOtion). The SMART system was equipped with eight TV cameras (100 Hz) with infrared light emitting diodes arranged in circular arrays concentric with the optical axes. The eight cameras were placed around the subject. Based on kinematic acquisition, spatiotemporal gait parameters were calculated for each trial. The parameters of interest were: speed (mm/s), cadence, (step/min), step length (mm), step width (mm), step length variability (Cov), and number of missteps during the tandem walk. The missteps were counted by looking at the recordings and curves of the markers on the feet. A misstep was defined as a step in which the whole foot was placed outside the tape the patients walked on. The other parameters were calculated using a Matlab program, i.e. m-Bape. The middle 80% of the recordings was used in order to eliminate the start-up and slow-down phases.

Postural evaluation

Postural control was analyzed using an AMTI force platform. The signals were acquired with a sampling frequency of 400 Hz. Centrer of pressure displacements (CoP) were calculated on the basis of these force recordings. Subjects stood on the platform for at least 15s in different stances, including 1) a natural position and 2) the Tandem Romberg position (feet in a heel-to-toe position) with their eyes open. Subjects were asked to stand as still as possible with their arms by their sides for at least 15s. Eight trials were collected for each subject in a random order.

In the natural condition, the CoP path corresponding to the distance covered by the successive positions of the CoP, was measured off-line. This index is classically used as an indication of the body sway velocity.

In the tandem condition, because many patients failed to maintain their posture for the required 15s, we developed a scale to assess the quality of postural control in this situation (Postural Evaluation for Tremor, PET). This scale (/6) took into account the total duration of the recording (score= duration/15x3) plus the number of times that subjects' balance was disturbed (0: unable

to perform the task, 1: at least 2 disturbances of balance, 2: 1 disturbance and 3: no disturbances). A score of 6/6 indicated that the patient had maintained the tandem position for at least 15 seconds without any imbalance. A score of 0 indicated that the patient was unable to perform the task.

Supplementary material 4. Speech and voice assessment before the radiosurgery of the 2nd side and 12 months after.

Gray cells and bold p-values refer to significant statistical comparison between pre- and post-surgery data.

A. Acoustics

**Tasks**<br>Measures	At GK2	12 Months after	p-value
Maximum sustained vowel /a/			
Max phonation time (in s)	15.4 (1.2-47.81)	16 (1.6-52.2)	0.681 ns
Oral diadochokinesis			
Articulatory rate<br>(= syllable number/speech duration, in syllables/second)	4.88 (3.07-6.86)	4.59 (1.80-6.45)	**0.020**
Speech proportion<br>(= total speech duration/total duration of the session, in s)	0.93 (0.69-0.99	0.94 (0.7-0.98)	0.726 ns
Pause proportion<br>(= pause duration/total duration of the session, in s)	0.07 (0.01-0.31)	0.06 (0.02-0.3)	0.734ns
Performance Index<br>(= speech proportion/number of breath groups)	0.16 (0.03-0.5)	0.19 (0.08-0.49)	0.616 ns
Breath duration (in s)	6.19 (0.98-15.8)	6.61 (2.43-16.77)	0.544 ns
Pause duration (in s)	0.47 (0.25-3.30)	0.47 (0.28-2.92)	0.902 ns
Number of syllables	27.4 (3-85.5)	13.75 (4.36-72)	0.959 ns
Sustained vowel /a/			
Mean fundamental frequency<br>(F0, in Hz)	137.84 (76.17-218.88)	141.63 (83.83-224.96)	0.245 ns
F0 SD (in Hz)	4.09 (0.92-52.4)	4.54 (0.86-21.2)	0.178 ns
Mean SPL (in dB)	73.99 (63.34-86.40)	73.05 (62.33-90.92)	0.483 ns
SPL SD (in dB)	1.47 (0.71-10.71)	1.73 (0.48-11.9)	0.382 ns
HNR	19.55 (3.5-25.2)	20.66 (9-34.3)	**0.035**
Jitter (in %)	0.006 (0.004-0.029)	0.006 (0.003-0.023)	0.492 ns
Shimmer (in Hz)	0.048 (0.19-0.2)	0.037 (0.012-0.16)	0.060 ns

Acoustic data were collected in quiet rooms in the neurology/neurosurgery wards of La Timone University Hospital (Marseille, France), using specialized equipment (Marantz PMD661 MKII recorder, USA; and a DPA 4288 CORE Directional Flex Headset microphone). The distance between the participant's mouth and the head-mounted microphone was kept consistent across participants to range from three to five centimeters. It was positioned at an angle of 45°-90° to the side of the lip angle, as recommended. The noise floor was not measured, but the experimental rooms were specifically chosen to prevent noise pollution from affecting the audio signals. All participants were instructed to perform speech tasks at a comfortable volume and

pitch to avoid straining their voices. Participants were recorded while performing non-speech movements to allow several acoustic parameters to be calculated:

(i) the maximally-sustained vowel /a/, for the calculation of the Maximum Phonation Time (MPT, s);

(ii) the production of a 3-second sustained vowel /a/, for the calculation of the mean fundamental frequency (F0, Hz) and its standard deviation (F0_SD, Hz), the sound pressure level (SPL, dB) and its standard deviation (SPL_SD, dB), the jitter (cycle to cycle F0 variation, %), the shimmer (cycle to cycle SPL variation, dB) and the Harmonics-to-noise ratio (HNR, %);

(iii) an oral diadochokinesis (ddk), namely the sequential motion rate task requiring the repetition of the tri-syllable pseudo-word /pataka/at the fastest rate possible during 30 seconds; the articulatory rate (in syllables/second), the speech and pause duration and proportions, as well as an oral ddk performance index (ddki = speech proportion/number of breath groups) were call.

B. Speech intelligibility and perceptual assessment

**Tasks**<br>Measures	At GK2	12 Months after	p-value
Speech intelligibility section of the Frenchay Dysarthria Assessment			
Sentences (intelligibility rate, in %)	100 (86,67-100)	100 (53,3-100)	0,754 ns
Words (intelligibility rate, in %)	96,7 (80-100)	93,3 (36,67-100)	**0.011**
Perceptual clinical evaluation (BECD)			
Phonetic realization	1.50	2.26	**0.02**

Speech intelligibility was rated following the instructions of the FDA-2 (1), which word and sentence sets were adapted into French (2) within the BECD (Batterie d'Evaluation Clinique de la Dysarthrie). The general procedure was as follows: ten words (out of 101) and ten sentences (out of 50) were randomly selected from the sets and presented to the participants, who read them aloud while being recorded. Off-line, three speech/language pathologists/therapists (SLP/Ts) rated the correct words and sentences. The scoring was: 0 = fewer than 5 words/sentences understood correctly; 1 = five to six words/sentences; 2 = seven to nine words/sentences; 3 = ten words/sentences understood with some effort; 4 = ten words/sentences easily understood. The speech intelligibility rates were calculated in percentages for the two types of stimuli (either words or sentences).

A speech task was also performed and recorded, as part of the audio recording protocol (see infra), and included reading aloud the French (3) version of Aesop's fable "*The North Wind and the Sun.*" Off-line, this allowed the three SLP/Ts to evaluate voice quality by using the perceptual score of the French Clinical Evaluation Battery of Dysarthria (*Batterie Clinique d'Evaluation de la Dysarthrie*, BECD) (4), which allows the assessment of six dimensions of voice and speech production across 35 items: voice quality (12 items), phonetic realization (6 items), prosody (12 items), respiration (3 items), intelligibility (1 item), naturalness (1 item).

Speech intelligibility and perceptual assessments were performed after the last post-operative examination of the last patient. The raters were blinded to the pre- or post-surgery status of the recordings, which were presented randomly.

C. Self-reported speech impairment questionnaires

**Tasks**<br>Measures	At GK2	12 Months after	p-value
Voice Handicap Index			
Total score	8.27 (0-56)	9.12 (0-64)	0.845 ns
Physical subscore	1.50 (0-15)	1.50 (0-18)	0.810 ns
Functional subscore	0.50 (0-25)	0.50 (0-18)	0.861 ns
Emotional subscore	0 (0-18)	0 (0-28)	0.972 ns
Dysarthria Impact Profile			
Total score	176.62	176.38	0.980 ns

Self-assessments were used to obtain information on the functional impact of the patient's speech/communication impairment and to provide patient-reported outcome measures (PROMs). A first questionnaire assessed the patients' self-evaluation of their voice and speech: the 30-item Voice Handicap Index - VHI-30 (5), adapted into European French (6).

The second one assessed the psychosocial impact of dysarthria: the Dysarthria Impact Profile - DIP (7), adapted into European French (8).

References

1. Enderby PM, Palmer R. FDA-2 : Frenchay Dysarthria Assessment. Second Edition. Pro-Ed (Firm). Austin, TX, USA.; 2008.

2. Ghio A, Giusti L, Blanc E, Pinto S. French adaptation of the "Frenchay Dysarthria Assessment 2" speech intelligibility test. European Annals of Otorhinolaryngology, Head and Neck Diseases. mars 2020;137(2):111-6.

3. Fougeron C, Smith CL. French. In: Handbook of the International Phonetic Association A guide to the use of the International Phonetic Alphabet. Cambridge University Press; 1999. p. 78- 81.

4. Auzou P, Rolland-Monnoury V. Batterie d'Evaluation Clinique de la Dysarthrie (BECD). Isbergues, France : Ortho Edition.; 2006.

5. Jacobson BH, Johnson A, Grywalski C, Silbergleit A, Jacobson G, Benninger MS, et al. The Voice Handicap Index (VHI): Development and Validation. Am J Speech Lang Pathol. août 1997;6(3):66-70.

6. Woisard V, Bodin S, Puech M. [The Voice Handicap Index: impact of the translation in French on the validation]. Rev Laryngol Otol Rhinol (Bord). 2004;125(5):307-12.

7. Walshe M, Peach RK, Miller N. Dysarthria Impact Profile: development of a scale to measure psychosocial effects. Int J Lang Commun Disord. janv 2009;44(5):693-715.

8. Atkinson-Clement C, Letanneux A, Baille G, Cuartero MC, Véron-Delor L, Robieux C, et al. Psychosocial Impact of Dysarthria: The Patient-Reported Outcome as Part of the Clinical Management. Neurodegener Dis. 2019;19(1):12-21.

Supplementary Material 5 : Efficacy parameters

Efficacy parameters at the time of the second radiosurgery and 12 months after the second radiosurgery.

		MEDIAN (IQR)	P-VALUE
TREMOR SEVERITY SCORE	Baseline	9 (7-10.25)	reference
	12 months	2 (1-5)	<0.001
FUNCTIONAL DISABILITY	Baseline	16.33 (9.80-18.20)	reference
	12 months	1.16 (0-4.37)	<0.001
ADL 2ND SIDE	Baseline	50 (43.93-57.91)	reference
	12 months	0 (0-7.76)	<0.001
AMPLITUDE 2ND SIDE	Baseline	20 (19-22)	reference
	12 months	6 (3-11.5)	<0.001
QUALITY OF LIFE (QUEST SCORE)	Baseline	35 (23.5-49)	reference
	12 months	13 (4.75-35)	<0.001

Bold indicate significant variable at p <0.05.
ADL : Activities of Daily Living
QUEST score : Quality of Life in essential Tremor Score

Clinical scores at baseline and 12-month follow-up after GK2. Boxplots represent median (horizontal line), interquartile range (box), and whiskers (1.5 * IQR). Individual patient data are overlaid as shaded circles with horizontal jitter. Light gray = Before GK; dark gray = 12 months after GK. *p < 0.05 (two-tailed Student's t-test). Exact p-values are reported for non-significant results.

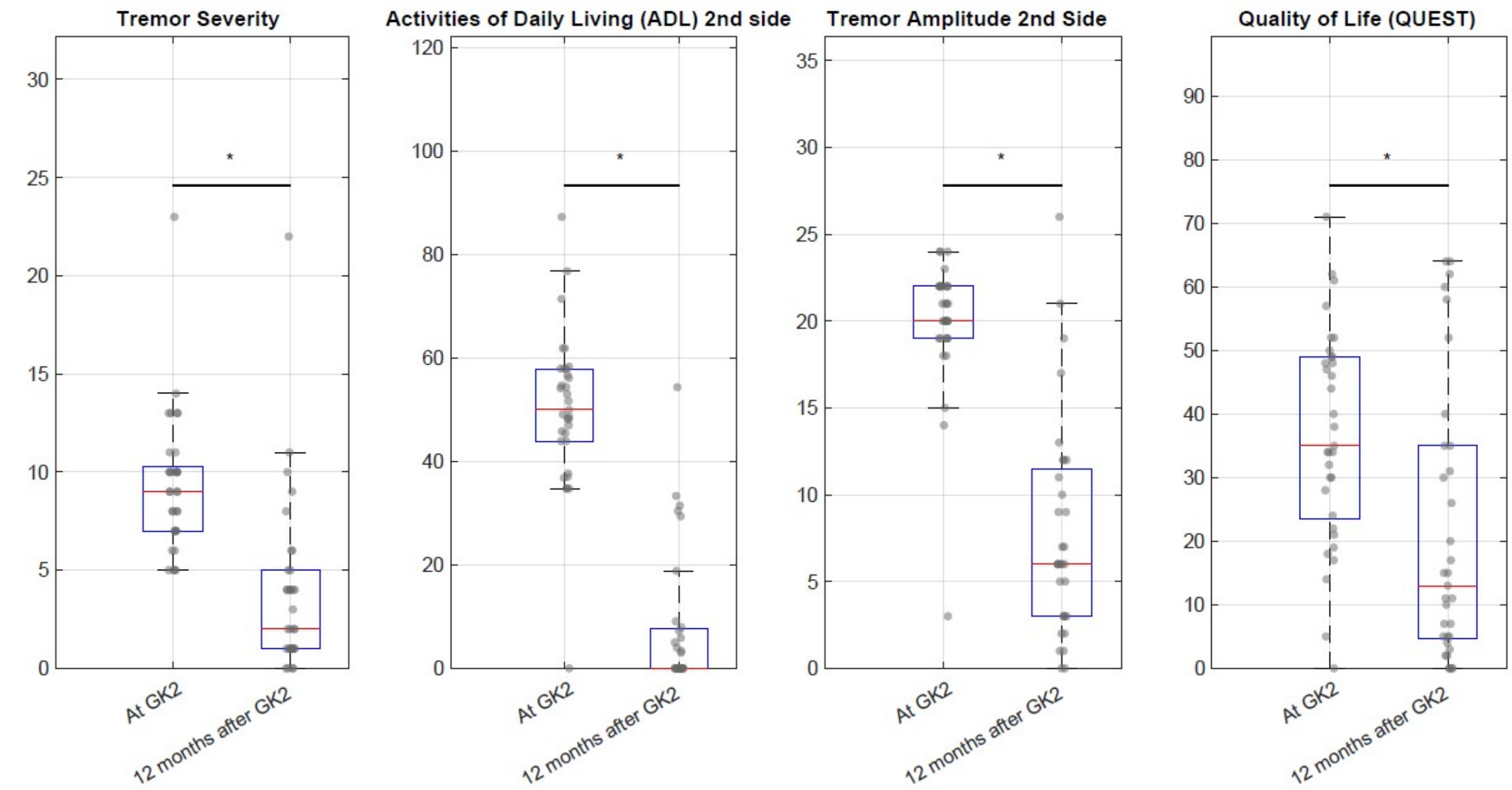

Supplementary Material 6 : MR image of the patient with the hyperresponse at 1 year post VIM-GKS

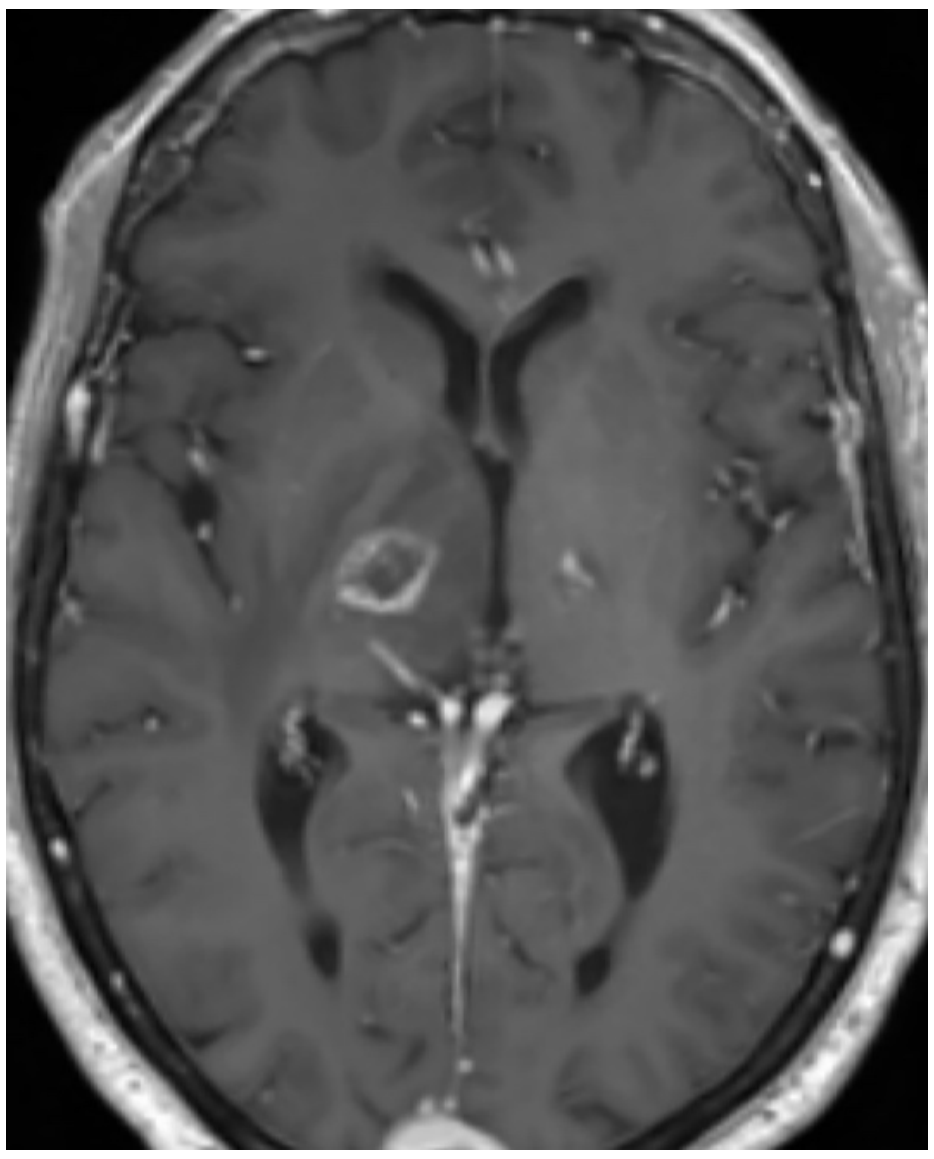

This image is displaying the large contrast enhancement area in the patient experiencing the hyper-response at one year after the VIM-GKS of the second side treated. Of note the small sequela of the first side treated VIM-GKS on the left side.

Supplementary Material 7 : Volume of the lesion on MR

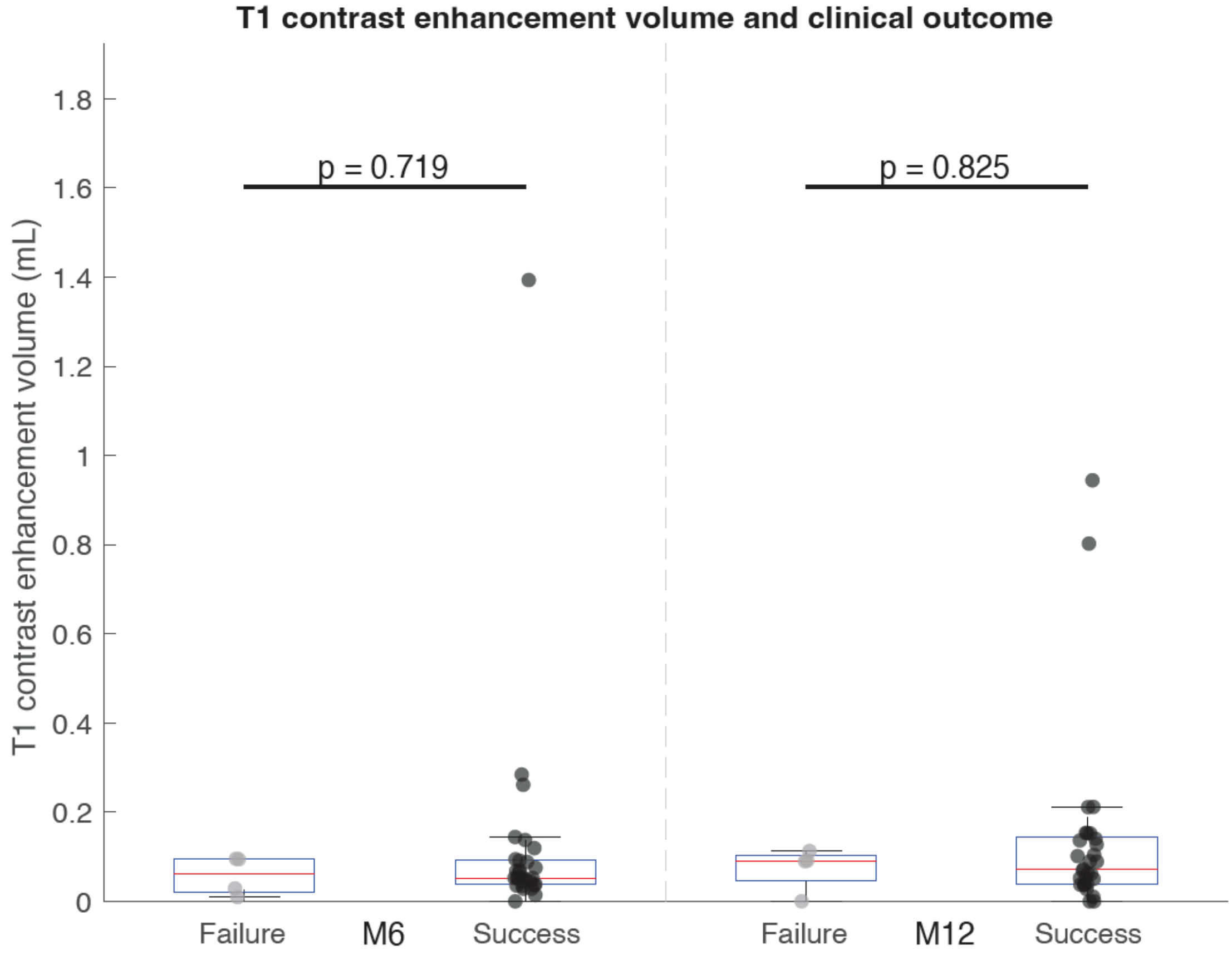

Relationship between T1 contrast enhancement volume and clinical outcome.
Boxplots showing T1 contrast enhancement volume on the side treated by the second VIM-GKS at 6 months (M6) and 12 months (M12), stratified by clinical outcome (Failure vs Success).
Boxes represent the median and interquartile range (IQR), with whiskers indicating minimum and maximum values. Individual data points are overlaid (Grey : Failure ; Black : Success). No statistically significant differences were observed between groups (M6: $p = 0.719$; M12: $p = 0.825$, Mann–Whitney U test).

Supplementary material 8: Description of the targeting and dose-planning.

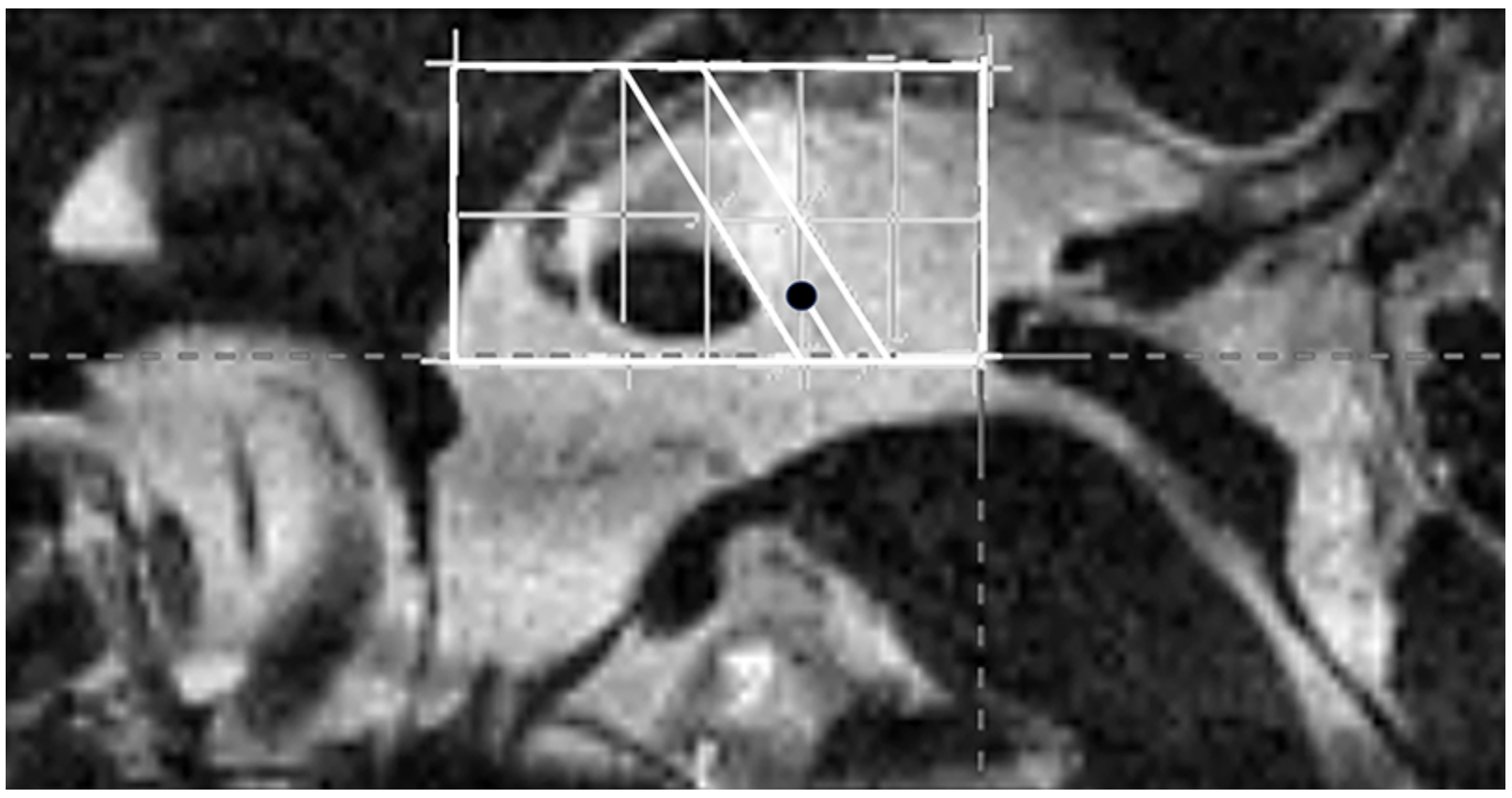

The VIM nucleus is targeted according to Gerard Guiot scheme

The radiosurgical target is placed 2,5 mm above AC-PC line (defined according to Guiot ventriculographic method with AC point at the postero-superior border of the anterior commissure and PC point at the antero-superior border of the posterior commissure).

Laterally the target is 11 mm from the lateral wall of the third ventricle. Then the pyramidal tract and the prelemniscal radiation are localized on the anisotropic fraction of the DTi sequence in order to check the lateral and inferior border of the VIM respectively. Finally the dento-rubro-thalamo-cortical tract tractography is used to confirm the target. A dose of 130 Gy is prescribed to the 100% isodose line of a 4 mm isocenter, using a dedicated shielding pattern to minimize radiation exposure to the internal capsule.

Author contributions

Jean Régis was the principal investigator of this prospective trial, contributed to the design of the study, performed all the operative procedures, contributed to the clinical follow up, the writing of the first draft of the manuscript with input from TW.

Marianne Vaugoyeau contributed to design the protocol of gait and posture evaluation.

Valentin Mira and Vaugoyeau Marianne did all the analyses of gait posture and balance and contributed to the writing of the manuscript.

Serge Pinto did all the analyze of speech and voice and contributed to the writing of the manuscript.

Jérémy Danna has worked on the acquisition of acoustic data, on their statistical analyses and interpretation and contributed to the writing of the section of the manuscript dedicated to the speech and voice.

Lauriane Veron-Delor has driven the perceptive evaluation (BECD, GRBAS) have worked on their statistical analyses and interpretation.

Eve Benchetrit did all the neuropsychological assessment and contributed to the writing of the manuscript.

Corentin Gaillard and Sarah Fernandes did the statistical analysis

Axelle Cretol was the data manager, contributed to the design and contributed to the writing of the manuscript.

Silvia Sandu contributed to the radiological follow up of the patients.

Jean Philippe Azulay contributed to the design of the study and the writing of the manuscript.

Alexandre Eusebio contributed to the design of the study and the writing of the manuscript.

Tatiana Witjas contributed to the design of the study, contributed to the clinical follow up, the interpretation of the data and the writing of the manuscript.

All the authors had full access to all the data in the study and had final responsibility for the decision to submit for publication.
